# Muscle fatigability and post‐acute COVID‐19 syndrome: A case study

**DOI:** 10.14814/phy2.15391

**Published:** 2022-08-18

**Authors:** Jacob Fanous, Alexander M. Zero, Charles L. Rice

**Affiliations:** ^1^ School of Kinesiology The University of Western Ontario London Ontario Canada; ^2^ Department of Anatomy and Cell Biology, Schulich School of Medicine and Dentistry The University of Western Ontario London Ontario Canada

**Keywords:** dorsiflexors, neuromuscular physiology, SARS‐CoV‐2 (COVID‐19), torque steadiness

## Abstract

The acute phase of COVID‐19 has been well studied, however with increasing post‐acute COVID‐19 syndrome, much is unknown about its long‐term effects. A common symptom in both the acute and post‐acute phases has been fatigue, assessed predominantly qualitatively. Here we present a case study objectively assessing neuromuscular fatiguability in a young male (27 year, 1.85 m, 78 kg) who continues to experience COVID‐19 related fatigue and cognitive dysfunction, including other symptoms, 12+ months post‐infection. Prior to infection, he was part of a neuromuscular study forming the basis of our pre‐COVID‐19 results. The study was repeated 12 months post‐COVID‐19 infection. Muscle strength, endurance, torque steadiness, voluntary activation, twitch properties, electromyography, and compound muscle action potential were obtained and compared pre‐ and post‐COVID‐19. All measurements were done using a dorsiflexion dynamometer in which the participant also was asked to produce a one‐minute fatiguing maximal voluntary contraction. Muscle strength, voluntary activation, and fatigability (slope of torque) showed no meaningful differences, suggesting intrinsic neuromuscular properties are not affected. However, torque steadiness was impaired three‐fold in the post‐ compared with pre‐COVID‐19 test. The participant also reported a higher level of perceived exertion subjectively and a continued complaint of fatigue. These findings indicate that muscle fatiguability in post‐acute COVID‐19 syndrome may not be a limitation of the muscle and its activation, but a perceptual disconnect caused by cognitive impairments relating to physical efforts. This case report suggests the potential value of larger studies designed to assess these features in post‐acute COVID‐19 syndrome.

## Graphical abstract

Post‐acute COVID‐19 syndrome has long lasting implications on the body, such as fatigue and cognitive dysfunction. When assessed objectively, fatigue in post‐acute COVID‐19 appears to be a limitation of cognition and not the muscle per se.

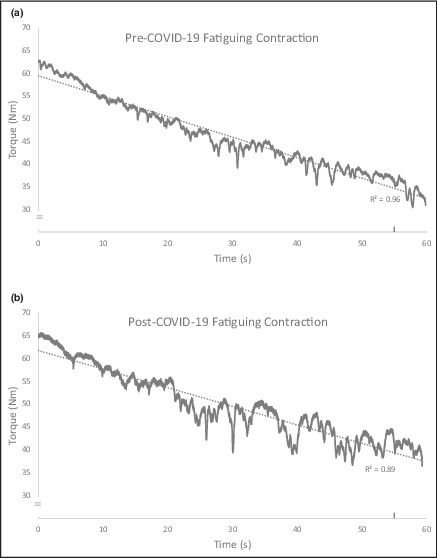

## 1 INTRODUCTION

In the acute phase, features of the severe acute respiratory syndrome coronavirus 2 (SARS‐CoV‐2) have been thoroughly described with clinical presentations such as respiratory and digestive complications, as well as myalgia (Huang et al., 2021; Wiersinga et al., 2020). However, long‐lasting complications of COVID‐19 have been less understood, but a frequent and debilitating symptom has been self‐reported fatigue, that may persist for at least 6–7 months after symptom onset (Yong, 2021). Other long‐lasting symptoms include dyspnoea, anosmia, and ageusia (Davis et al., 2021; Jason et al., 2021; Yong, 2021), as well as cognitive dysfunction, colloquially termed brain fog (Deumer et al., 2021; Murga et al., 2021), which involves reduced performance on attention tasks, working memory, and problem solving (Hampshire et al., 2021).

Fatigue is a broad term that can encompass multiple definitions (Enoka & Duchateau, 2008). In a clinical setting, fatigue has been defined as a subjective sense of tiredness with a lack of physical and mental energy (Krupp et al., 1988; Lerdal et al., 2009), and is the more common definition used to describe general fatigue associated with COVID‐19. This differs from the physiological definition that relates to neuromuscular fatigue, which is the reduction in expected muscle contractile force during the performance of a specific task and can be objectively measured (Bigland‐Ritchie et al., 1995) and induced using electrically evoked or voluntary contractions. Given the limitation of not being able to test a subject infected with COVID‐19 prior to their illness onset, most literature pertaining to fatigue in COVID‐19 has focused on the clinical definition which has been subjective and survey‐related (Deumer et al., 2021; Huang et al., 2021; Tabacof et al., 2022).

Here we present a case report of a young male patient who has post‐acute COVID‐19 syndrome with symptoms lasting over a year post‐infection. Fortuitously, this patient had participated in a neuromuscular fatigue study approximately 1 month prior to contracting COVID‐19. The participant was then reassessed using the same fatiguing protocol 12 months after the acute phase of COVID‐19, thus providing a unique opportunity to compare objective and quantifiable measures of neuromuscular fatiguability in a laboratory setting. As a graduate trainee in the lab, the participant was well‐familiarized with these laboratory tests over the previous several years.

Although, the patient did not require hospitalization for the acute phase, qualitatively he reported severe illness including intense general fatigue, high fever, muscle aches, and shortness of breath which led to being bedridden for 6 days after testing positive with a polymerase chain reaction (PCR) test for the COVID‐19 virus. Furthermore, at the time of data collection for the post‐COVID‐19 results (12 months post‐acute infection) the participant continued to experience persistent symptoms, including general fatigue, cognitive dysfunction such as trouble concentrating, shortness of breath, and significant loss in smell and taste. All of which confirms a case of post‐acute COVID‐19 syndrome.

## 2 METHODS

### 2.1 Case presentation

The participant was a 27‐year‐old male who was infected with COVID‐19 in the fall of 2020. This was supported with a positive PCR test and various classic COVID‐19 symptoms. The participant reported having severe symptoms during his infection and was almost admitted to the hospital. He experienced anosmia (loss of smell) and ageusia (loss of taste) during and post‐acute infection, which continues to persist with minimal improvement more than 12 months later. Additionally, he described having severe brain fog that slowly improved over the year, although at this time he still complains of general fatigue, loss of appetite, and insomnia. He was first tested, considered as the pre‐COVID‐19 results, in the late summer of 2020 and the post‐COVID‐19 testing occurred in the early fall of 2021.Participant provided oral and written consent prior to testing. Study procedures were approved by the local research ethics review board for Health Science Research Involving Human Participants (no. 107505), and conformed to the standards set by the Declaration of Helsinki, except for registration in database.

### 2.2 Experimental set‐up

To assess neuromuscular properties, the participant was seated upright on a chair with the left leg placed in a custom‐built isometric dorsiflexor dynamometer to record torque output. Both knee and hip joints were positioned at 90 degrees, and the ankle positioned at 110 degrees plantar flexion (Marsh et al., 1981). The foot was tightly secured with straps and a metal C‐bracket was placed firmly over the anterior thigh to limit hip and knee flexion. Dorsiflexor torque was transmitted through a footplate and strain gauge located at the axis of the ankle joint and the output was analog to digital converted (Power 1401, Cambridge Electronic Design), and sampled at 500 Hz (Spike2, Cambridge Electronic Design). Visual feedback of torque was provided in real‐time using a monitor.

To record global neural activity, electromyography (EMG) surface electrodes were placed in a monopolar arrangement with the active electrode on the muscle belly of the tibialis anterior (TA) and the reference on the distal TA tendon. A ground electrode was placed on the lateral malleolus. Surface EMG signals were sampled at 2 kHz (Spike2, Cambridge Electronic Design), pre‐amplified (100x), and filtered between 10 Hz and 2500 Hz (Neurolog, NL844, Digitimer).

### 2.3 Electrical stimulation

Electrical stimulation was used to evoke contractile responses of the dorsiflexors. To elicit muscle twitch responses from the dorsiflexors a standard clinical stimulating bar electrode connected to a stimulator (ModelDS7AH; Digitimer) was placed over the common fibular nerve just inferior and posterior to head of the fibula. For stimulation, a square wave pulse duration of 200 μs was delivered at 400 V. Current intensity was increased until the compound muscle action potential (CMAP) reached a plateau (40 mA). For the fibular nerve and tibialis anterior muscle, this resulted also in a plateau of dorsiflexion twitch torque. The current subsequently was increased by 20% to a supramaximal level for testing procedures.

### 2.4 Protocol

Twitch responses from the dorsiflexors were first determined and then the participant was asked to produce a 2.5 s maximum voluntary contraction (MVC) of the dorsiflexors to familiarize him with producing MVCs. After at least 5‐min rest period the participant performed another MVC, and the interpolated twitch technique (ITT) was used to assess voluntary activation (VA) and this was repeated a second time. The ITT involved eliciting a muscle twitch at rest prior to the MVC, followed by a 2.5 s MVC with a twitch induced at the plateau of the MVC contraction (interpolated), and subsequently, another twitch was induced immediately post MVC with the muscle at rest. All twitches were evoked using a single pulse stimulus at 100 μs. The ITT protocol was quantified as: VA = 1 – (interpolated twitch torque/post‐MVC twitch torque) × 100 (Todd et al., 2004).

After another 5‐min rest period, a voluntary fatiguing task was performed consisting of a one‐minute sustained dorsiflexor MVC. Voluntary muscle activation was assessed at 55 s by superimposing a twitch which was compared using the ITT method with the twitch delivered immediately after the fatiguing task when the muscle was at rest.

### 2.5 Data analysis

Analyses were performed offline in Spike 2 (Cambridge Electronic Design). Peak torque was determined for all evoked and voluntary contractions, which are defined as the maximal value in Nm. The isometric MVC was the peak torque value during the plateau of the maximal contraction done prior to the fatiguing task. Supramaximal muscle twitches contractile properties of peak torque (Nm), maximal rate of torque development (RTD) in Nm/s, time‐to‐peak tension (ms), and one‐half relaxation time (ms) were measured at baseline (pre‐fatigue).

Surface electromyography (EMG) was used to assess global muscle activity of the TA muscle. During baseline dorsiflexion MVCs, root‐mean‐squared (RMS) amplitude was measured during a 1‐s epoch at the contraction plateau. During the one‐minute fatiguing task RMS was calculated from a 1‐s epoch at 5, 15, 30, 45, and 55 s. These values were normalized to the baseline CMAP peak‐to‐peak amplitude. The difference between the normalized RMS value during the task were compared across the two different test sessions. At baseline and during the fatiguing task at 55 s, VA was assessed using the ITT technique as noted above.

In R (version 4.1.2), torque steadiness was assessed during the one‐minute fatiguing MVC by assessing the sum of squares error (SSE) of the torque tracing to the line of best fit. The torque tracing was extracted from Spike 2 and consisted of ~30,000 points across the entire 60 s.

## 3 RESULTS

Height and weight of the subject did not change between the two (pre and post‐COVID‐19) testing sessions (Table 1), however, the subject reported a loss of ~5 kg in the initial 3 months after being infected due to reduced food intake from dyspepsia and lack of physical activity.

**TABLE 1:** Participant characteristics

Measurement	Pre‐COVID‐19	Post‐COVID‐19
Age	26 years	27 years
Mass	80 kg	78 kg
Height	1.85 m	1.85 m
BMI	23.4 kg/m^2^	22.8 kg/m^2^

Abbreviation: BMI, body mass index.

No meaningful differences were found in MVC torque, voluntary activation, twitch parameters, or CMAP amplitude (Table 2).

**TABLE 2:** Baseline

Measurement	Pre‐COVID‐19	Post‐COVID‐19
MVC	64.5 Nm	67.7 Nm
Voluntary activation	98.8%	97.9%
Peak twitch torque	9.5 Nm	11.1 Nm
Twitch maximal RTD	24.9 Nm/s	28.8 Nm/s
Twitch one‐half‐relaxation time	86.8 ms	78.5 ms
CMAP peak‐peak amplitude	11.8 mV	12.0 mV

Abbreviations: CMAP, compound muscle action potential; RTD, rate of torque development.

**FIGURE 1:**
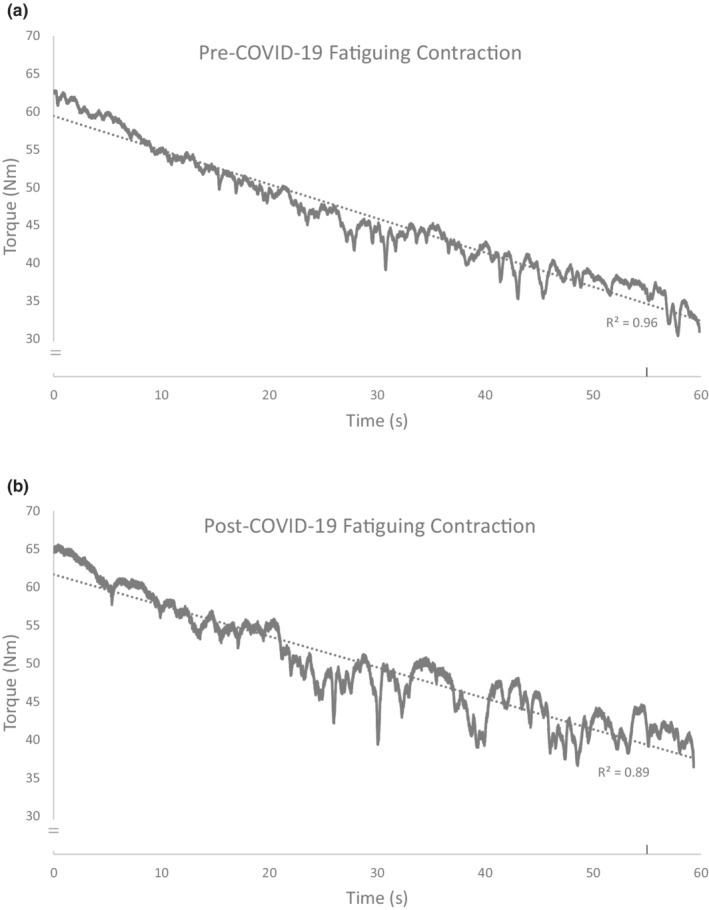
Torque trajectories of the fatiguing maximal voluntary dorsiflexion contractions held for 1 min. Rise to peak torque at time 0 s (from 0 Nm to maximal torque value) and end of contraction at time 60 s (to 0 Nm) are not included in the lines of fit (dotted lines). (a) Contraction pre‐COVID‐19 session. (b) Contraction post‐COVID‐19 session. Note: The reduction in torque steadiness in the post‐COVID‐19 contraction. Vertical tick mark on *x*‐axis denotes stimulation for the superimposed twitch used to assess voluntary activation.

During the 1‐min fatiguing contraction, no physiological difference in mean relative EMG activity (mean difference 0.13%) or voluntary activation were found (Table 3). The rate of torque decline was relatively similar between the two testing sessions (Table 3), with a slope difference of 0.05 Nm/s (Figure 1). However, the line of best fit throughout the torque tracing of the fatiguing task indicated that torque steadiness in the second testing session (post‐COVID‐19) was lower, with an *R* ^2^ values of 0.89, versus 0.96 pre‐COVID‐19, respectively. This was further quantified by the error calculation using the standard deviation around the line of best fit, and the torque steadiness was three times worse (greater fluctuations in torque tracing) in the post‐COVID‐19 fatiguing task than in the first (pre‐COVID‐19) session.

**TABLE 3:** Fatiguing contraction

Time (s)	Measurement	Pre‐COVID‐19	Post‐COVID‐19
5	Torque	62.5 Nm	65.7 Nm
15	Torque	52.9 Nm	55.3 Nm
30	Torque	43.6 Nm	46.7 Nm
45	Torque	41.0 Nm	41.1 Nm
55	Torque	39.1 Nm	42.0 Nm
	Voluntary activation	92.4%	94.6%
	CMAP amplitude	9.0 mV	8.9 mV

Abbreviation: CMAP, compound muscle action potential.

## 4 DISCUSSION

This case provides unique observations and insights regarding the implications of muscle fatiguability in post‐acute COVID‐19 syndrome. We were able to objectively assess neuromuscular properties including isometric strength and endurance, voluntary activation, surface EMG, and torque steadiness in a young adult male experiencing persistent COVID‐19 symptoms 12 months post‐infection.

Despite population level self‐reported persistence of fatigue with post‐acute COVID‐19 (Agergaard et al., 2021), we did not detect any change in dorsiflexor isometric strength, voluntary activation, CMAP and EMG parameters, or voluntary muscle fatigability in our participants 1‐year post COVID‐19. These results would indicate there is no direct effect on key neuromuscular factors despite the on‐going feeling of fatigue. Thus, there may be a disconnect between perceptual general fatigue and the intrinsic ability of the neuromuscular system to achieve and maintain torque in a fatiguing task involving maximal muscle effort contractions. However, and importantly, the participant reported that maintaining torque during the task was much more difficult, denoted as a higher sense of effort (Revill & Fuglevand, 2017), and that performance was worse. This was reflected in the torque record during the task which was less gradual and smooth (reduced steadiness) in the second session (post) compared with the first session (pre).

Some studies have raised the possibility that COVID‐19 may be causing chronic fatigue syndrome (CFS) in post‐acute COVID‐19 and thus leading to the observed fatigue complications (Deumer et al., 2021; Murga et al., 2021), but in this case, there was no reduction in actual muscle torque nor a decrease in voluntary activation calculated using the twitch interpolated technique (Todd et al., 2004). A decrease in both is a major hallmark for CFS (Sacco et al., 1999). Additionally, there were no changes in neural drive to the muscles as assessed by similar relative EMG activity, CMAP amplitude, and voluntary activation. Although the sense of effort was greater (not objectively quantified) pain sensitivity subjectively reported was not changed. Agergaard et al. (2021) also noted no significant difference in CMAP parameters in their sample of COVID‐19 subjects, but they did report that intramuscular EMG measures were suggestive of myopathic changes. With no change in absolute strength or endurance capacity we have no evidence of any possible myopathic changes, however, it could be one contributing factor to the change in steadiness that requires further investigation. Indeed, the combination of these measures and observations in this one participant supports that there may be other factors, differing from CFS, causing the reported general sense of activity‐dependent fatigue and feeling of weakness. This could indicate that central factors related to cognitive functions (brain fog) or perception have been altered.

Given the results, we postulate that it may be a limitation in cognitive function, experienced by those with post‐acute COVID‐19 syndrome that is contributing to many debilitating symptoms, including reports of fatigue. The data presented here would suggest that muscle mass (given a similar body weight), absolute isometric strength, and endurance ability (fatigability) of the dorsiflexor leg muscles during a maximal isometric fatiguing task are not directly affected, but a change in the sense of effort may be, and should be included as an objective measure in future studies. This notion is further supported by research on olfaction. A report by Lechien et al. (2020) showed that ~40% of COVID‐19 patients that have self‐reported loss of smell were not actually anosmic, when tested objectively using laboratory measures. This discrepancy in self‐reports and objective testing of anosmia also was reported by Vaira et al. (2020). Moreover, Oaklander et al. (2022), have shown that small‐fiber neuropathy, affecting sensory/autonomic axons, was commonly found in those with prolonged post‐acute COVID‐19 syndrome. Afferent fibers may therefore be affecting the feedback between muscle output and sense of effort leading to an increase in perceived difficulty (i.e. reduced torque steadiness) despite the frank lack of any neuromuscular limitations. Assessing objective neuromuscular properties, in conjunction with cognitive function and afferent feedback, may help to elucidate factors contributing to fatigue symptoms reported by COVID‐19 patients and direct treatment therapies.

## FUNDING INFORMATION

The study was supported by NSERC to C. L. Rice.

## CONFLICT OF INTEREST

The authors have no conflicts of interest to report.
